# Supplementary material for: Content-rich biological network constructed by mining PubMed abstracts
Source: BMC Bioinformatics. 2004 Oct 8;5:147. doi: 10.1186/1471-2105-5-147 (PMC528731; doi:10.1186/1471-2105-5-147)
Supplement: Additional File 2 — The original results of the above study (non-essential files are deleted to keep the file size under the limit set by BMC bioinformatics). [file 1471-2105-5-147-S2.bz2 › chilibotAdditionalFile2/dip30/145ID8955331E515/html/RSBV_RSBW.html]

 


 **RSBV** and **RSBW** 
  
Found 25 abstracts in PubMed, retrieved 25.  
 

 What does Google say? 
 PDF only 
| .edu only 

---

**Interactive relationship** (e.g. stimulation, inhibition, etc)

**Inhibitory relationship**- Inhibition of sigma B by  **RsbW**  is counteracted by  **RsbV** , a protein that is essential for the activation of sigma B dependent transcription.  Ref: 8144446 J Bacteriol, 1994
- **RsbV**  reversed this inhibition by sequestering  **RsbW**  in a  **RsbW**   **RsbV**  complex, thereby allowing sigma B to remain free and active.  Ref: 8764398 J Mol Biol, 1996
- In confirmation and extension of previous results, we show that  **RsbW**  is also a protein kinase that uses ATP to phosphorylate  **RsbV** , thereby blocking the capacity of  **RsbV**  to bind to  **RsbW**  and activate transcription.  Ref: 8764398 J Mol Biol, 1996
- The activity of sigma B is controlled in part by  **RsbW** , a protein that inhibits sigma B, and  **RsbV** , a protein that counteracts this inhibition.  Ref: 8764398 J Mol Biol, 1996
- sigma B dependent transcription is induced by the activation of sigma B itself, a process that involves release of sigma B from an inhibitory complex with its primary regulator,  **RsbW** . sigma B becomes available to RNA polymerase when  **RsbW**  forms a complex with an additional regulatory protein  **RsbV**  and, because of this, fails to bind sigma B.  Ref: 7601843 J Bacteriol, 1995
**Neutral relationship**- In contrast to interactions among the components of the homologous regulatory system for the sporulation transcription factor sigma F, the binding of  **RsbW**  to  **RsbV**  and sigma B did not require adenosine nucleotides.  Ref: 8764398 J Mol Biol, 1996
- Interactions between a Bacillus subtilis anti sigma factor  **RsbW**  and its antagonist  **RsbV** .  Ref: 8144446 J Bacteriol, 1994
- The phosphorylated  **RsbV**  species comigrated during IEF with the  **RsbW**  dependent form of  **RsbV**  found in crude cell extracts.  Ref: 8144446 J Bacteriol, 1994
- On the basis of this genetic analysis, our principal conclusions are that i the rsbS product is a negative regulator of or activity, ii the rsbT product is a positive regulator, iii RsbS requires RsbT for function, and iv the RsbS RsbT and  **RsbV**   **RsbW**  pairs act hierarchically by a common mechanism in which key protein protein interactions are controlled by phosphorylation events.  Ref: 8682789 J Bacteriol, 1996
- **RsbW**  is also a protein kinase which can phosphorylate  **RsbV** .  Ref: 12270815 J Bacteriol, 2002
- When gel filtration fractions containing  **RsbV**   **RsbW**  complexes or  **RsbV**  alone were subjected to IEF, only the unmodified form of  **RsbV**  was found associated with  **RsbW** .  Ref: 8144446 J Bacteriol, 1994
- However, following stress,  **RsbW**  binds instead to the  **RsbV**  anti anti sigma factor, thereby releasing sigma B to direct transcription of its target genes.  Ref: 8682789 J Bacteriol, 1996
- In vitro,  **RsbV**  became phosphorylated when incubated with ATP and  **RsbW**  but not with ATP alone.  Ref: 8144446 J Bacteriol, 1994
- The yeast reporter system was activated when RsbS was paired with either RsbR or RsbT, RsbR was paired with RsbT, and  **RsbV**  was paired with either RsbU or  **RsbW** .  Ref: 8955331 J Bacteriol, 1996
- Two forms of  **RsbV**  were visualized when crude cell extracts of B. subtilis were subjected to isoelectric focusing IEF, with the more negatively charged  **RsbV**  species absent from extracts prepared from  **RsbW**  strains.  Ref: 8144446 J Bacteriol, 1994
- We now demonstrate that purified  **RsbW**  is capable of forming alternative complexes with either sigma B or  **RsbV** .  Ref: 8764398 J Mol Biol, 1996
- When cells are stressed,  **RsbW**  binds to unphosphorylated  **RsbV** , produced from the phosphorylated form of  **RsbV**  by two phosphatases RsbU and RsbP which are activated by stress.  Ref: 12270815 J Bacteriol, 2002
- When crude B. subtilis extracts were fractionated by gel filtration chromatography or electrophoresis through nondenaturing polyacrylamide gels, a complex composed of  **RsbW**  and  **RsbV**  that is distinct from the previously observed  **RsbW**  sigma B complex was detected.  Ref: 8144446 J Bacteriol, 1994
- According to the current understanding of sigmaB regulation, in unstressed cells the phosphorylated  **RsbV**  anti anti sigma is unable to complex the  **RsbW**  anti sigma, which is then free to bind and inactivate sigmaB.  Ref: 10632888 Mol Microbiol, 2000
- **RsbW** , purified as a dimer, forms complexes with  **RsbV**  and sigma B with different stoichiometries, i.e.,  **RsbW**  2  **RsbV**  2 and  **RsbW**  2 sigma B 1.  Ref: 12270815 J Bacteriol, 2002
- Low levels of ATP cause  **RsbW**  to release sigma B and bind to an alternative protein  **RsbV** , while high levels of ATP favor  **RsbW**  sigma B complex formation and inactivation of  **RsbV**  by an  **RsbW**  dependent phosphorylation.  Ref: 8808936 J Bacteriol, 1996
- Notably, the predicted rsbS product has significant amino acid identity to the  **RsbV**  anti anti sigma factor and the predicted rsbT product resembles the  **RsbW**  anti sigma factor.  Ref: 8682789 J Bacteriol, 1996
- We conclude that the higher affinity of  **RsbW**  for  **RsbV**  than for sigma B, rather than a difference in the concentrations of  **RsbV**  and sigma B, is the driving force that is responsible for the switch of  **RsbW**  to unphosphorylated  **RsbV** .  Ref: 12270815 J Bacteriol, 2002
- Experiments involving the exchange of proteins between the two regulatory systems demonstrated that  **RsbW**  and its homolog in the sigma F system, SpoIIAB, exhibit strong preference in binding to  **RsbV**  and sigma B, and SpoIIAA and sigma F, respectively,.  Ref: 8764398 J Mol Biol, 1996

**Non-interactive relationship** (e.g. studied together, co-existance, homology, etc.)

- These two principal regulators of sigmaB activity are encoded in the eight gene sigB operon, which has the gene order rsbR rsbS rsbT rsbU  **rsbV**   **rsbW**  sig B rsbX where rsb stands for regulator of sigma B.  Ref: 8682789 J Bacteriol, 1996
- As determined by surface plasmon resonance, the dissociation constants of the  **RsbW**   **RsbV**  and  **RsbW**  sigma B interactions were found to be similar 63 and 92 nM, respectively.  Ref: 12270815 J Bacteriol, 2002
- The sigB operon of Bacillus subtilis encodes sigma B plus three additional proteins  **RsbV** ,  **RsbW** , and RsbX that regulate sigma B activity.  Ref: 8468294 J Bacteriol, 1993
- The intracellular concentrations of  **RsbV** ,  **RsbW**  as a monomer, and sigma B measured before stress were similar 1..6, and 0.9 micro M, respectively.  Ref: 12270815 J Bacteriol, 2002
- **RsbW**  and  **RsbV**  have been shown to control sigma B activity, functioning as an inhibitory sigma B binding protein and its antagonist, respectively.  Ref: 8002609 J Bacteriol, 1995
- Three genes  **rsbV** ,  **rsbW** , and rsbX, which are cotranscribed with the sigma B structural gene sigB, encode regulators of sigma B dependent gene expression.  Ref: 8002609 J Bacteriol, 1995
- Evidence for gene identification includes the following i phylogenetic analyses of reported amino acid sequences for sigmaB and the closely related sigmaF proteins grouped L. monocytogenes sigmaB in the same cluster with the sigmaB proteins from Bacillus subtilis and Staphylococcus aureus, ii the gene order in the 68 bp portion of the L. monocytogenes sigB operon is rsbU  **rsbV**   **rsbW**  sigB rsbX and is therefore identical to the order of the last five genes of the B. subtilis sigB operon, and iii an L. monocytogenes sigmaB mutant had reduced resistance to acid stress in comparison with its isogenic parent strain.  Ref: 9658010 J Bacteriol, 1998
- The deduced amino acid sequences of orf2, orf3, orf4, and orf5 show 6 1, and 77% similarity to the B. subtilis proteins RsbU,  **RsbV** ,  **RsbW** , and SigB, respectively, with SigB representing the sigma factor and the Rsb proteins representing regulators of sigma B.  Ref: 9042755 Arch Microbiol< MedlineTA>Arch Microbiol, 1998
- The increase was greatest for  **RsbV** , whose concentration reached 13 micro M, while those of  **RsbW**  as a monomer and sigma B reached 11.8 and 4.9 micro M, respectively.  Ref: 12270815 J Bacteriol, 2002
- and v both the  **rsbV**  and  **rsbW**  products appear to regulate sigma B activity posttranslationally.  Ref: 1592822 J Bacteriol, 1992
- In analogous experiments, RsbX, an additional regulator of sigma B dependent transcription that is thought to act independently of  **RsbV**   **RsbW** , was not found to associate with any of the other sigB operon products.  Ref: 8144446 J Bacteriol, 1994
- The results are consistent with RsbU, or a process dependent on RsbU, being able to facilitate the  **RsbV**  dependent release of sigma B from  **RsbW**  but normally prevented from doing this by RsbX.  Ref: 8002609 J Bacteriol, 1995
- A close correlation was observed between the concentration of ATP required for efficient  **RsbW**  mediated phosphorylation of  **RsbV** , inhibition of  **RsbW** . **RsbV**  comlex formation, and inhibition of sigma B directed transcription.  Ref: 8764398 J Mol Biol, 1996
- sigma B activity is regulated post translationally by a multi component network composed of two coupled partner switching modules, RsbX RsbS RsbT and RsbU  **RsbV**   **RsbW** , each containing a serine phosphatase X or U, an antagonist protein S or V, and a switch protein serine kinase T or W.  Ref: 9179850 Mol Microbiol, 1997
- Three genes  **rsbV** ,  **rsbW** , and rsbX, cotranscribed with the sigma B structural gene sigB, regulate sigma B dependent gene expression.  Ref: 8460143 Proc Natl Acad Sci U S A, 1993
- We show that this network comprises two partner switching modules, RsbX RsbS RsbT and RsbU  **RsbV**   **RsbW** , which contribute to regulating sigma B.  Ref: 8824586 Genes Dev, 1996
- Environmental stress induces the release of sigma B and the formation of the  **RsbW**   **RsbV**  complex, regardless of ATP levels.  Ref: 8808936 J Bacteriol, 1996
- iv the rsbX,  **rsbV** , and  **rsbW**  products have a hierarchical order of action.  Ref: 1592822 J Bacteriol, 1992
- The operon encoding the general stress transcription factor sigma B and two proteins of its regulatory network,  **RsbV**  and  **RsbW** , was cloned from the gram positive bacterium Bacillus anthracis by PCR amplification of chromosomal DNA with degenerate primers, by inverse PCR, and by direct cloning.  Ref: 10960085 J Bacteriol, 2000
- Genetic data and in vivo measurements argue that RsbX negatively regulates an extension of the  **RsbV**   **RsbW**  pathway that requires the product of an additional regulatory gene rsbU which lies immediately upstream of the sigB operon.  Ref: 8002609 J Bacteriol, 1995
- These two classes of stress are transmitted by separate signaling pathways which converge on the direct regulators of sigma B, the  **RsbV**  anti anti sigma factor and the  **RsbW**  anti sigma factor.  Ref: 11591687 J Bacteriol, 2001
- Biochemical techniques previously revealed physical associations among  **RsbW** ,  **RsbV** , and sigmaB but failed to detect interactions of RsbR, S, T, U, or X with each other or  **RsbV** ,  **RsbW** , or sigmaB.  Ref: 8955331 J Bacteriol, 1996
- Bracketing slr1860 icfG are i ORF slr1861, whose predicted product resembles the SpoIIAB, RsbT, and  **RsbW**  protein kinases from B. subtilis, and ii ORFs slr1856 and slr1859, whose predicted products resemble the respective phosphoprotein substrates for the B. subtilis protein kinases SpoIIAA, RsbS, and  **RsbV** .  Ref: 10438742 J Bacteriol, 1999
- Nonetheless, an analysis of the complexes by nondenaturing polyacrylamide gel electrophoresis in competition assays suggested that the affinity of  **RsbW**  2 for  **RsbV**  is much higher than that for sigma B.  Ref: 12270815 J Bacteriol, 2002
- There are likely to be at least two routes by which information can enter the system to regulate sigma B via the rsbX product, and via the  **rsbV**  and  **rsbW**  products.  Ref: 1592822 J Bacteriol, 1992
- Using an anti sigma B monoclonal antibody to monitor the levels of sigma B protein, PSPAC to control the expression of the sigB operon, and a ctc lacZ reporter system to monitor sigma B activity, we observed that the  **rsbV**  and  **rsbW**  products control sigma B activity at the ctc promoter independently of their effects on sigma B levels.  Ref: 8468294 J Bacteriol, 1993
- The polypeptides encoded by orf33 0 59, and 256 showed 6 1, and 77% homologies, respectively, with the RsbU,  **RsbV** ,  **RsbW** , and SigB polypeptides encoded by the B. subtilis sigB operon.  Ref: 8830703 J Bacteriol, 1996
- This pathway requires the products of additional genes encoded within the eight gene operon sigB that includes the genes for sigma B,  **RsbW** , and  **RsbV** .  Ref: 8808936 J Bacteriol, 1996
- The presumed phosphorylated variant of  **RsbV**  was present only in fractions that did not contain  **RsbW** .  Ref: 8144446 J Bacteriol, 1994
- The complete sigB operon is preceded by a sigma A like promoter PA and has the order PA orfR orfS orfT orfU PB  **rsbV**   **rsbW**  sig B rsbX, where rsb stands for regulator of sigma B and the previously identified sigma B dependent promoter PB is an internal promoter preceding the downstream four gene cluster.  Ref: 8002610 J Bacteriol, 1995
- Using Western blot immunoblot analyses, reporter gene fusion assays, and measurements of nucleotide pool sizes, we provide evidence for two independent processes that promote the binding of  **RsbW**  to  **RsbV** .  Ref: 7601843 J Bacteriol, 1995
- Western blot immunoblot analyses, in which monoclonal antibodies specific for the sigB operon products  **RsbV** ,  **RsbW** , and sigma B were used to probe extracts from wild type and mutant B. subtilis strains, revealed that all three proteins increased coordinately after heat shock.  Ref: 8458834 J Bacteriol, 1993
- The data are consistent with  **RsbV**  and  **RsbW**  being regulators of sigma B activity and RsbX acting primarily as a negative regulator of sigB operon expression.  Ref: 8468294 J Bacteriol, 1993
- In other experiments, Western blot immunoblot analyses showed that the levels of RsbR, RsbS, Rsb, and RsbU, unlike those of the sigB operon s four downstream gene products  **RsbV** ,  **RsbW** , RsbX and sigma B, are not elevated during sigma B activation.  Ref: 8682769 J Bacteriol, 1996
